# Socio-demographic factors influencing the ownership and utilization of insecticide-treated bed nets among malaria vulnerable groups in the Buea Health District, Cameroon

**DOI:** 10.1186/1756-0500-7-624

**Published:** 2014-09-10

**Authors:** Helen Kuokuo Kimbi, Sarah Bi Nkesa, Judith Lum Ndamukong-Nyanga, Irene Ule Ngole Sumbele, Julius Atashili, Mary Bi Suh Atanga

**Affiliations:** Department of Zoology and Animal Physiology, Faculty of Science, University of Buea, PO Box 63, Buea, SWR Cameroon; Department of Public Health and Hygiene, Faculty of Health Sciences, University of Buea, PO Box 63, Buea, SWR Cameroon; Department of Nursing, Faculty of Health Sciences, University of Bamenda, Bamenda, NWR Cameroon

**Keywords:** Malaria, Prevention, Bed nets, Pregnant women, Children, Cameroon

## Abstract

**Background:**

Malaria remains a public health problem and the use of insecticide-treated bed nets (ITNs) against it in vulnerable groups (pregnant women and children <5 years) is recommended in Cameroon. This study was aimed at assessing the socio-demographic factors influencing the ownership and utilization of ITNs among vulnerable groups in the Buea Health District (BHD).

**Methods:**

In a cross-sectional survey a questionnaire was administered in households with at least a child <5 years and/pregnant woman in five health areas of the BHD. Information on demographic variables, household composition, mosquito bed net (MBN) ownership, utilization and factors influencing ownership and utilization was recorded.

**Results:**

A total of 443 respondents were recruited and 208 (47.0%) possessed at least one MBN (total = 275 MBNs) with a median of 1.33 nets. Of the 275 nets found in households, 89 (32%) were potent ITNs and others had never been retreated/treated. Purchase of MBNs from the market was associated with marital status (*P* = 0.010) and urban settlement (*P* = 0.045). The number of respondents who did not know where to retreat/treat ITNs was significantly higher (*P* = 0.005) in urban than rural dwellers. The proportion of rural respondents who had once taken their MBNs for re-treatment was significantly higher (*P* = 0.002) than that of urban dwellers. MBN utilisation was 69.7% (95% confidence interval; CI = .63.2–75.6%). A total of 83.4%, 13.8% and 3.4% used MBNs throughout the year, during the rainy and dry seasons respectively. MBN use in children under five was associated with being from an urban area (*P* = 0.01). MBN use in pregnant women was associated with living in block-louver houses than in block-pane houses (*P* = 0.047).

**Conclusions:**

Utilization of MBN needs to be encouraged to match ownership while free distribution of ITNs to vulnerable groups needs to be continuous and consistent.

**Electronic supplementary material:**

The online version of this article (doi:10.1186/1756-0500-7-624) contains supplementary material, which is available to authorized users.

## Background

Malaria remains a public health problem in sub-Saharan Africa and it is more serious in vulnerable groups such as children under five years old and pregnant women [1]. Malaria transmission is perennial in all regions of Cameroon including the BHD where almost all citizens fall ill with it each year [2]. The National Malaria Control Program (NMCP) report of activities shows that in the South West Region of Cameroon, 56% of consultations, 54% of hospital admissions and 53% of deaths among children below five years are due to malaria [3]. Similarly, 42% of consultations, 70% of hospital admissions, and 12% of deaths among pregnant women are due to malaria.

The crumbling effects of malaria on the vulnerable groups and the socio-economic development of the nation can be greatly reduced through prevention. The main strategies of malaria prevention in Cameroon are intermittent preventive treatment (IPT) for pregnant women and vector control through the use of ITNs especially for pregnant women and children under five [3]. Freely distributed ITNs have been in existence in households in Cameroon since 2003 following the Abuja Declaration in 2000. The main objective of this initiative was to prevent transmission, infection and suffering among the vulnerable populations – children under five years and pregnant women [4]. In February 2007, long lasting insecticide treated bed nets (LLINs) were distributed to households with at least one child under five years of age in the BHD [5]. ITNs are also being distributed in the country free of charge to pregnant women during antenatal clinics (ANC) while the rest of the community members obtain their own ITNs from the regional treatment units (RTU) and community treatment units (CTUs) where ITNs are re-impregnated with insecticides after regular intervals of six months by community relay agents (CRA) who have been trained to carry out this exercise. Unfortunately, ITN ownership and utilization appear to be in disparity as analysed by the Malaria Report for Cameroon [6]. It would seem that this difference between ownership and utilization is related to the level of knowledge on malaria and socio-demographic factors as age and marital status [7, 8] as well as education and occupation. Elsewhere, the gap between ownership and utilization of bed nets has also been shown to be wide [9–11].

Kimbi *et al.* [12] reported that malaria morbidity is still relatively high in some parts of the BHD. Again, a household survey of malaria indicators conducted in Obala, Cameroon, showed that 80% of households were in possession of MBNs, 73.41% of children under five slept under such nets and fever was reported in children under five in 75% of households [13]. However, this survey did not report on the utilization of ITNs among vulnerable groups and the factors influencing such utilization. Takem *et al.* [14] reported a 16.95% ITN use in Buea, but the study did not differentiate between ITN use by vulnerable groups and the general population. Therefore, little is known about the actual ITN ownership, utilization and knowledge of the factors determining the utilization of ITNs by pregnant women and children under five in the BHD. Against this background this study was aimed at assessing the socio-demographic factors influencing the ownership and utilization of ITNs among malaria vulnerable groups in the BHD, Cameroon.

## Methods

### Study area

The study was conducted in the BHD in August, 2011. Buea is the capital of the South West Region of Cameroon and is situated on the eastern slopes of Mount Cameroon. Buea Health District has a population of 133,092 inhabitants and an estimated 10,259 households. The BHD is partitioned into seven health areas for the purpose of community health activities. The climate of Buea generally, is of the equatorial type with temperatures ranging from 18°C–29°C annually with average humidity of 80%. There are two main seasons; the rainy season which starts from mid-March to November and the dry season which lasts for the rest of the year. Human malaria is meso-endemic during the dry season but becomes hyper-endemic in the rainy season, with incidence peaking in July-October. The prevalence of malaria parasitaemia in the Mount Cameroon region varies from 60.6%, in low land altitude to 7.7% in the highlands [15].

### Study population

The study participants were pregnant women and mothers/care-givers of under-fives in selected households in five of the seven Health Areas. A total of 443 participants were recruited including 139 pregnant women.

### Study design

The study was a descriptive cross-sectional design. A pre-tested questionnaire and an observational checklist were used as data collection tools. To ensure proper administration of the questionnaire, a team of 6 interviewers was trained by the researchers for two days before the start of data collection on the tools to be used, purpose of the study and how to approach respondents and obtain consent. Data was collected by face to face interviews of respondents. The questionnaire was divided into four main sections. Section A of the questionnaire concentrated on demographic variables which included health area, community of residence, type of settlement (rural or urban), age, sex, level of education, marital status, religion and profession, household composition and house construction materials. Section B was designed to enable the collection of data concerning ownership of MBNs, sources of owned MBNs and insecticidal status of the MBNs. Finally, section C assessed the utilization of the MBNs, problems associated with the utilization and the influence of community relay agents (CRAs) on the use of MBNs. An additional file shows the questionnaire used in detail (see Additional file 1). The checklist enabled the interviewer to verify the number of MBNs in the household, if they were hung over the bed and their physical state (whole or torn).

The first thing in each eligible household was to identify the mother of an under-five or a pregnant woman. In cases where the mother could not be met on two different visits, the father or eldest mature household member (at least 15 years) was recruited as the respondent.

### Sampling method

A multistage sampling method was used for this study. The BHD consists of seven health areas made up of sixty-seven communities. Some are rural while others are urban. Five health areas were randomly selected; Bokwango, Buea Road, Bova, Tole and Muea. From each of the five areas chosen, six communities were randomly chosen. In each randomly selected community, households with at least one pregnant woman/or a child under five years were visited. The number of households to be visited for each health area and for each community was determined by considering the proportion of the population of the health area/community to the total population under study. The following procedure was used to select each household: Firstly, members of the research team stood at the centre of each street in the community and a direction was randomly chosen by spinning a bottle and the direction of the head of the bottle was chosen. Depending on the number of households found in the direction of the bottle head, each household with a pregnant woman and or child under five was visited. At the end of the direction, if the target was not attained, the team members returned to the starting point and took the opposite direction and followed the same technique until the target for the community was reached.

### Ethical considerations

An ethical clearance for this study was obtained from the University of Buea Faculty of Health Sciences Institutional Ethical Committee. In addition, an administrative authorization was obtained from the Regional Delegation of Public Health, South West Region. The traditional chiefs of the various communities were also contacted and their authorizations obtained before entry into the communities. Potential respondents gave verbal consent after they had been given an explicit explanation of the study and an opportunity to ask and respond to any questions.

### Case definitions for the study

For clarity of concepts, the following terms were defined as:

*Household:* refers to all the persons living in the same house, sharing the same meal and who recognize the authority of a head known as the household head.

*Insecticide-treated bed net*: refers to a bed net that has been impregnated with an insecticide (short duration not more than six months and long lasting not more than five years).

*Mosquito bed net:* refers to any bed net (treated/untreated/unspecified).

*Ownership*: is the proportion of households in possession of at least a bed net at the time of the study.

*Utilization:* refers to the proportion of households that own a MBN under which all children under five and all pregnant women slept the night preceding the interview.

### Data management and analysis

Data collected were entered into Epi info, version 3.5.1 (WHO/CDC, Atlanta, USA) and backed-up in MS Excel 4.0 (Microsoft Inc, Seattle, USA). It was then transferred to STATA 9 (STATA Corps, Texas, USA) for cleaning and analysis. Uni-variate analysis was done using simple frequency calculations and medians. Bi-variate analysis and comparisons of proportions between groups were done with the chi square (*χ*^2^) and Fisher’s exact tests (F). Multivariate analysis was done by performing logistic regressions. The different categories of settlement areas, type of respondents, level of education, marital status, income status, locality and settlement type were entered into a logistic regression model as independent variables to assess the factors associated with the purchase of MBN from the market and utilization of MBNs among the children less than 5 years and pregnant women. In addition, other variables such as the level of knowledge of malaria and the type of windows in their homes were included as independent variables in the models evaluating factors associated with MBN utilization. Significant levels were measured at 95% confidence level with significant differences recorded at P <0.05.

## Results

### Demographic characteristics of participants

Of the 443 participants recruited 139 (31.4%) were pregnant women, 290 (65.5%) were urban dwellers and the age ranged from 15 to 73 years with a median of 28 years. The majority were mothers of under-fives (54.4%, 241) while the least were elder brothers/sisters of the under-fives (3.8%, 17) as shown in Table 1.

**Table 1:** **Demographic characteristics of participants**

Variable	Category	Number	%
Settlement	Rural	153	65
	Urban	290	35
Respondent	Mother	380	86
	Father	46	10
	Brother/Sister	17	4
Median age in years (Interquartile range)	All	28 (15–73)	
Sex	Female	394	89
	Male	49	11
Level of education	None	22	5
	Primary	159	36
	Secondary	142	32
	High school	65	15
	University	55	12
Marital status	Married	292	66
	Single	137	31
	Widowed	10	2.28
	Separated	3	0.68
Religion	Christians	438	99
	Muslims	5	1
Profession	House wives	80	18
	Business	66	15
	Students	66	15
	Farming	53	12
	Tailoring	44	10
	Teaching	40	9
	Hair dressing	27	6
	Health personnel	18	4
	Others	49	11

### Ownership, sources and types of MBNs in households

Of the 443 households interviewed, 208 (47.0%) were in possession of at least one MBN (total = 275 MBNs) with a median of 1.33 MBNs in each household. Only 118 (27%) admitted knowing the price of MBNs in the market. The greatest number of MBNs in households (136/275, 49%) were obtained from ANC, while 26 (9%), 45 (16%) and 68 (26%) were gotten from health campaigns, market and school/NGO/Red Cross/relatives respectively.

In all, of the 275 MBNs found in the households, only 89 (32%) were potent ITNs because all the others had never been retreated/treated or had expired.

A regression analysis for the purchase of MBNs from the market with the various demographic variables (after adjusting for all variables) revealed a strong association between marital status (OR = 3.6, P = *P* = 0.010; 95% CI = 1.37–9.77) and urban settlement (OR = 4.49, *P* = 0.045, 95% CI = 1.03–19.57) as shown in (Table 2).

**Table 2:** **Association of demographic factors of participants with the purchase of MBNs from the market**

Demographic category	OR	***P***-value	95% CI
Caretaker of under-five	1		
Mother/pregnant woman	1.51	0.68	0.22–10.50
13-20 years	3.48	0.21	0.49–24.80
21-50 years	0.96	0.96	0.17–5.54
51+ years	1		
Female	0.29	0.23	0.04–2.23
Male	1		
Low level education	1		
High level education	0.89	0.77	0.43–1.87
Married	3.66	0.010	1.37–9.77
Single	1		
Non income earners	1		
Income earners	1.21	0.60	0.60–2.45
Rural	1		
Urban	4.49	0.045	1.03–19.57
Bokwango	1		
Buea road	0.55	0.36	0.15–1.99
Muea	0.97	0.96	0.28–3.37
Tole	1.43	0.72	0.21–9.91

### Awareness about the community treatment unit (CTU)

Majority of the respondents (304, 69%) did not know where their nets could be treated/retreated and the proportion was significantly higher (*χ*^2^ = 7.84, *P* = 0.005) in urban (36%) than rural dwellers (23%). Of the 139 (31%) respondents who knew where their CTU was located, only 36 (26%) had taken a net there for re-treatment. The proportion of rural respondents (46%, 16) who had once taken their MBNs for re-treatment was significantly higher (*χ*^2^ = 9.57, *P* = 0.002) than that of urban dwellers (19%, 20%).

### Social and personal factors that influenced the utilization of MBNs

Of the 208 households that owned MBNs, 145 (69.7%, CI = 63.2–75.6%) reported utilization of the MBNs. The utilization of MBNs by the under-fives was highest in plank-pane (59, 29.0%) and lowest in plank-louver houses (7, 19%) while a higher proportion of households with plank-louver houses (12, 29%) than brick-pane (1, 11%) had all pregnant women sleep under MBNs.

Reported barriers to the use of MBNs were poverty, colour and shape of MBN, feeling uncomfortable under a MBN and much time required for the usage of the net. Of the 145 respondents reporting to be using MBNs, 121 (83.4%) used MBNs throughout the year while 20 (13.8%) and 5 (3.4%) used MBNs only in the rainy season and dry season respectively.

Table 3 shows the association between demographic factors and the utilization of MBNs in children less than five and pregnant women. After adjusting for other measured variables indicated in the table, the factors significantly associated with the use of MBNs in children under five included being from an urban area (OR = 2.44, 95% CI: 1.44–4.13; *P* = 0.01). Children who were significantly less likely to use MBNs were those from the Buea Road health area (OR = 0.35, 95% CI: 0.13–0.95, *P* = 0.039) and of health personnel (OR = .0.11, 95% CI: 0.01–0.93, *P* = 0.04).

**Table 3:** **Association of demographic factors with the utilization of MBNs among children less than five years and pregnant women**

Demographic factor	Category	OR	p-value	95% CI	OR	p-value	95% CI
		Children under five years			Pregnant women		
Area	Bokwango	1			1		
	Bova	0.67	0.52	0.19–2.30	7.06	0.048	1.02–48.90
	Buea Road	0.35	0.04	0.13–0.95	0.58	0.58	0.82–4.07
	Muea	1.16	0.75	0.45–3.06	4.83	0.09	0.79–29.48
	Tole	0.77	0.68	0.22–2.63	17.29	0.01	1.80–165.88
Respondent	Caretaker of Under-fives	1			1		
	Mothers	0.71	0.65	0.16–3.06	0.05	0.89	0.001–1.55
Age (years)	13-20	4.57	0.18	0.48–43.65	0.67	0.47	0.23–1.99
	21-50	7.17	0.07	0.84–60.88	1		
	≥51	1			6.70	0.01	1.46–30.64
Sex	Male	1			1		
	Female	3.01	0.19	0.58–15.68	1		
Level of education	Low level education	0.83	0.53	0.48–1.46	0.16	0.002	0.05–0.52
	High level education	1			1		
Marital status	Married	1.38	0.31	0.75–2.55	6.70	0.01	1.46–30.64
	Single	1			1		
Income status	Non-income earners	1			1		
	Income earners (excluding health personnel)	0.62	0.08	0.37–1.05	0.16	0.002	0.05–0.52
	Health personnel	0.11	0.04	0.01–0.93	0.49	0.33	0.12–2.00
Window type	Block Pane	1			1		
	Block Louvers	1.57	0.38	0.57–4.29	5.99	0.04	1.08–33.29
	Plank Pane	2.21	0.13	0.80–6.08	11.23	0.12	0.54–234.50
	Plank louvers	1.37	0.63	0.38–4.97	13.92	0.10	0.62–310.57
Level of Malaria knowledge	Low	1			1		
	High	0.86	0.69	0.40–1.84	0.49	0.33	0.12–2.00
Settlement type	Rural	1					
	Urban	2.44	0.01	1.44–4.13	5.99	0.04	1.08–33.29

After adjusting for all demographic variables, pregnant women were 7.06 times more likely to sleep under MBNs in Bova (*P* = .0.048, 95% CI =1.02–48.90) and 17.29 times in Tole (*P* = 0.013, 95% CI: 1.80–165.88) than those in Bokwango. Also, married pregnant women were 6.69 times more likely to sleep under MBNs when compared with single pregnant women (*P* = 0.014, 95% CI = 1.46–30.64). After adjusting for all other factors, pregnant women living in block-louver houses were 20.67 times more likely to sleep under MBN than those living in block-pane houses (*P* = 0.047; 95% CI = 1.04–409.9). When all other demographic variables were controlled a strong evidence of association (*P* = 0.002, 95% CI: 0.052–0.524) was observed in the use of MBNs between income earning pregnant women and non income earners. Pregnant women who earned some income were 0.16 times more likely to use the MBNs than their non-income counterpart (Table 3). After adjusting for every other factor, pregnant women in urban communities were 5.99 times more likely to sleep under MBNs than those in rural communities and the difference was statistically significant (*P* = 0.041, 95% CI: 1.078–33.29).

### Household observations

When observed only 97% (267) MBNs were actually seen in homes. The rest could not be easily brought out from their preserved positions or had been given out. Sixty-two (35%) of those hung over the bed had holes and tears. The MBNs that were not hung over the beds were seen in different states: 2 (3%) torn beyond use, 1 (0.6%) was hung on the windows, 38 (49%) were just folded and lying somewhere in the room and 36 (47%) were still sealed inside their packages.

## Discussion

This study examined the socio-demographic factors influencing the ownership and utilization of MBNs in the Buea Health District. Overall, only 47% of households interviewed owned at least one MBN. This coverage is far less than the coverage of 80% recorded in Obala, Cameroon in 2009 [13]. This may be due to the fact that the study in Obala was done just two years after the distribution campaign and many people still had their nets intact. The majority of the nets distributed in BHD in 2007 might have been torn and out of use at the time of this study. This is supported by the fact that a good proportion of nets observed in homes were torn and out of use. It is also worth noting that the free distribution of ITNs was done in the different regions in Cameroon at different times, for example, those for the East, South and South West Regions, were in 2007 while those for the West and Littoral Regions were in 2009. Furthermore, residents of the North West, Centre and East Regions continue to receive free LLINs from other partners such as PLAN Cameroon (a non-governmental organisation). Tobin-West and Alex-Hart [16] also reported a 37.2% utilization in Nigeria and the main reason for this low rate was the tropical heat associated with the use of bed nets.

The results showed that the majority of MBNs were obtained from ANC and during immunization campaigns. Only 16% of the MBNs found in households were purchased from the market. Many respondents did not even know the price of a MBN in the market as respondents apparently had the attitude of receiving free MBNs. This implies that cost of MBNs was certainly an important barrier to the ownership of bed nets in this area. One of the ways to ensure ownership and use of these MBNs will be for the government to provide them free of charge. At worst the government could subsidize the prices thus making them more accessible and affordable to the poor who form the majority of the population in the study area. A similar situation was reported in Nigeria by Singh and Singh [17].

The practice of purchasing MBNs from the market, ownership and utilisation of MBNs were found to be associated with marital status. The married respondents were 3.66 times more likely to buy a MBN from the market when compared with the single respondents. This can be linked to the fact that married women received financial aid from their husbands unlike single women who struggle on their own to take care of all family responsibilities. Generally, better malaria prevention and control strategies including MBN use have been associated with financial or socio-economic status of the individuals concerned as they are directly linked to accessibility and affordability of the preventive measures [18].

Respondents in the urban areas were significantly more likely to purchase MBNs from the market than those in the rural communities. Generally, urban dwellers are wealthier and more educated than those in the rural communities. Studies by Legesse *et al.* [19] associated education and wealth status with comprehensive knowledge on malaria preventive measures. This probably explains why respondents from urban areas were better able to buy MBNs from the market. It is worth noting that the free distribution of ITNs at health facilities had halted at the time of this study, such that only a few pregnant women reported having received a MBN for the current pregnancy. This inconsistency in the program can be a source of laxity on usage even for those who are in possession of MBNs. The attention of the health authorities and stakeholders in the malaria control program has to be drawn to this inconsistency in the free distribution of ITNs especially to vulnerable groups as the failure of the program could result in high rates of malaria morbidity and mortality among them.

The utilization of MBNs/ITNs by children under five and pregnant women lagged behind ownership. This corroborates the findings of Afolabi *et al.* [20] who reported high possessions of ITNs and low utilization in Nigeria. Tsuang *et al.* [21] postulated that in such situations, many household members may be forced to make difficult decisions about who should sleep without the protection of the MBN. This implies that great proportions of the vulnerable groups were not protected against malaria with ITNs or were partially covered by untreated or expired MBNs. This potentially placed the high risk groups in particular and other members of the households in general at higher risk due to mosquito “diversion effect”. This agrees with the reports of Lines *et al.* [22] and Tsuang *et al.* [21]. This implies that the burden of malaria morbidity and mortality among the vulnerable groups of BHD may continue to exist and even increase. Interestingly, malaria accounted for 7% of post-neonatal child deaths globally in 2010 and 15% of post-neonatal child deaths in Africa [23]. This is astounding for a disease that is preventable and treatable. Afolabi *et al.* [20] declared that the use of ITNs remains one of the best cost-effective interventions against malaria. They are estimated to be twice as effective as untreated MBNs and offer 70% protection when compared with no net at all [24]. Contrary to ownership of MBNs by vulnerable groups that was linked to urban settlement the reverse was the case with knowledge of treatment centres and the use of MBNs. This may be due to the fact that people in the rural areas are less able to afford malaria treatment and therefore take preventive measures more seriously than their urban counterparts.

The low utilization of ITNs by under-fives (38%) was however, higher than the 13% reported for 2006 in Cameroon [25] before the free distribution of LLINs to households with under-fives in BHD. Parents of those who own MBNs therefore need to be encouraged to put them into use in order to achieve the optimum target. This relatively low utilization is probably due largely to socio-demographic and personal factors and not due to lack of MBNs in households because not all the households that had MBNs put them into use. Some MBNs were found still sealed inside their packages, while some were hung on windows. This phenomenon is common in many sub-Saharan countries [20, 26]. Perhaps, with the low level of education, in some cases the respondents do not appreciate why they should use MBNs or how to hang and set them up. It is worth noting that ITN use has been shown to reduce clinical malaria episodes by approximately 50% and all-cause mortality by 17% [27]. In situations where ITN coverage in the community is above 60%, a community effect has been seen in which non-users receive similar protection to ITN users [20]. Reducing malaria burden therefore will likely contribute to the attainment of the millennium development goals, especially those related to reduction in malaria deaths and poverty, while improving maternal and infant health.

Respondents’ level of awareness of treatment centres was very low and out of the 103 (23%) who knew where their treatment centres were found, only 36 (26%) of them had ever taken a MBN there for treatment. The population needs to be sensitized on the location of treatment centres and the necessity to treat purchased ordinary MBNs especially those used over babies’ cots. This could best be done by training health personnel who will be responsible for giving out information on bed net use during ANC and infant welfare activities at health facilities. Community relay agents could be motivated to give extensive information, education and communication campaign messages to the households in ‘Pidgin’ English and or local languages to advocate for a change in behaviour. The local community radio stations and television channels could be used where well “structured” messages on bed net use in relation to a reduction in malaria incidence/morbidity could be broadcast in such languages [12, 28].

In the effort to use MBNs, respondents reported a number of challenges. These barriers could be overcome by adopting the door-to-door distribution and hang up strategies employed by the Alliance for Malaria Prevention, the President’s Malaria initiative and the International Federation of Red Cross and Red Crescent Society, as described by the United States Agency for International Development [29].

The preferred colour of the MBN was controversial. The green colour was the most uncomfortable colour for some, yet, very comfortable for others. The blue colour was the most comfortable. The green colour was however preferred by some people because it tolerates dirt and stains more than bright colours such as white and sky-blue. These findings agree with those of Ng’ang’a *et al.* [30]. The rectangular bed net was also preferred by most (74%) of the respondents because it is more spacious while the conical net is easier to mount, more convenient for baby’s cot and for small rooms. Therefore, colour preference and shape of MBNs remain very subjective and depend to a large extent on the individuals concerned.

In the present study the utilization of MBNs by children in block houses was lower than that of children in plank houses; thus children in block houses were more at risk of mosquito bites and consequently malaria infections. This high level of utilization of nets in plank-louver houses would probably result in fewer episodes of malaria of the occupants of such houses in this study. Community members need to be sensitized to know that mosquitoes get into the houses through the door and not only through the windows and cracks on the walls and therefore proper and consistent use of nets will reduce the number of malaria episodes in the net users and by extension other household non-net members as suggested by Tsuang *et al.* [21].

One of the major strengths of this work is the fact that it was conducted during the peak rainy season when the majority of respondents reported using MBNs and this period coincided with the peak malaria transmission season. Elsewhere, the cold rainy season has been reported to be a key reason for using MBNs [31, 32]. Some of the challenges of the study include the fact that there was no established list of households in any of the health areas and so systematic simple random selection of households was a little difficult. Our instrument was not sensitive enough to observe household members actually sleeping under the MBNs at night. Therefore, actual utilization may be lower than reported.

## Conclusions

The utilization of bed nets was lower than ownership. There is therefore a need to develop new strategies for sensitization messages to reach every community member. The NMCP should ensure that the free distribution of ITNs to vulnerable groups is continuous and consistent in the BHD. Community members need to be sensitized to purchase ordinary bed nets and take them for impregnation at the CTUs and not only wait for free distributions. CRAs activities also need to be updated to involve hanging of nets in homes and helping household members to overcome social and personal barriers in the use of bed nets.

## Authors’ information

HKK: PhD and Associate Professor of Medical Parasitology, Head of Department Zoology and Animal Physiology

SBN: MPh in Public Health

JLN-N: MSc and Assistant Lecturer of Zoology

IUNS: PhD and Lecturer of Parasitology

JA: PhD and Lecturer of Public Health

MBSA: PhD and Lecturer of Nursing, Head of Department, Nursing

## Electronic supplementary material

Additional file 1: **Questionnaire.** (PDF 74 KB)

## Abbreviations

ANC: Antenatal clinics
BHD: Buea Health District
CRAs: Community relay agents
CTUs: Community treatment units
ITNs: Insecticide-treated bed nets
LLINs: Long lasting insecticide treated bed nets
MBN: Mosquito bed net
NMCP: National Malaria Control Program
RTU: Regional treatment units.
