# Supplementary material for: Socio-demographic factors influencing the ownership and utilization of insecticide-treated bed nets among malaria vulnerable groups in the Buea Health District, Cameroon
Source: BMC Res Notes. 2014 Sep 10;7:624. doi: 10.1186/1756-0500-7-624 (PMC4167508; doi:10.1186/1756-0500-7-624)
Supplement: Supplementary file 1 — Additional file 1: Questionnaire. (PDF 74 KB) [file 13104_2014_3153_MOESM1_ESM.pdf]

QUESTIONNAIRE ON

SOCIO-DEMOGRAPHIC FACTORS INFLUENCING THE UTILIZATION OF INSECTICIDE TREATED BED NETS AMONG VULNERABLE - GROUPS IN BUEA HEALTH DISTRICT

We are researchers from the University of Buea, carrying out a study on the above subject. We would be grateful if you will give complete and honest responses to the following questions. Your responses and the observations we make shall be kept strictly confidential and shall be used solely for the purpose of this research. If you have mosquito nets at home, we would like to see them and also take photographs of them just where they are in your room.

A. DEMOGRAPHIC DATA

1. Respondent ID No. __________________________ **2.** Phone No: ____________________

3. Health Area_________________________________**4.** Quarter/Community____________

5. Community Settlement: **1. Rural 2. Urban** **6.** Tribe_________________________

7. Responsibility of Respondent: 1. **Father**

2. **Mother**

3. **Eldest Sister/Brother**

8. Age____**9.** Sex: **1. Male 2. Female**

**10.** Level of Education:	**11.** Marital Status	**12.** Religion
1. **None**	1. **Married**	1. **Christian**
2. **Primary**	2**. Single**	2. **Moslem**
3. **Secondary**	3. **Widow/Widower**	3. **Others**

4. **High school** 4. **Separated**

5. **University** 5. **Divorced**

13. Profession____

14. Total number of people in the household___________________________

15. Number of children under five years in the household____________________

16. Number of pregnant women in the household________________________

17. Type of housing construction material:

1. Plank **2. Blocks**

18. Type of window construction:

1**. Louvers** **2. Pane**

B. KNOWLEDGE, ATTITUDES AND PRACTICES TOWARDS MALARIA, ITS TRANSMISSION AND PREVENTION

19. Have you ever heard of malaria?

1. **Yes** 2. **No**

20. If yes to question 19 above from whom**?**

1. **Radio** 2.**Television** 3.**Health Facility**

4. **Community Relay Agent (CRA)** 5. **Tracts/posters** 6**.Others**

21. What does malaria mean to you? ____________________________________

22. How would you know if someone has malaria? __________________________

23. How many of your household members have presented with signs of malaria during the past four months: a) Children less than five years? _______________________

b) Pregnant women? ____________________________________

24. What are the effects of malaria in a child less than five years? _______________

____

25. What are the effects of malaria in a pregnant woman? ________________________

____

26. Malaria is a dangerous disease, what do you say?

1. Strongly disagree 2. Disagree 3. Neutral 4. Agree 5. Strongly agree

27. If you agree to 26 above, what makes malaria a dangerous disease? ______________

____

28. How can someone get malaria? ____

____

29. How can one prevent malaria? ____

____

C. AWARENESS, KNOWLEDGE AND POSSESSION OF MOSQUITO BED NETS

30. Have you heard of insecticide treated bednets?

1. **Yes** 2. **No**

31. If yes to question 30 above, from whom?

1. **Radio** 2**.Television** 3**.Health unit** 4**. CRA** 5**.Tract**

6. **Others___________________________**

32. What do you think of mosquito bednets as a preventive measure against malaria?

1. **Very bad** 2. **Bad** 3**. Good** 4.**Very good** 5. **Excellent**

33. What in your opinion are the benefits from using mosquito bednets? _____________

____

34. In your culture or religion, are there any prohibitions against sleeping under bednets?

1. **Yes** 2. **No.**

35. If yes to question 34 above, what ill will befall you if you sleep under a bednet?____

____

36. What other reasons might prevent people from sleeping under a mosquito bednet?

____

____

37. Do you have any mosquito bednet in your house?

1. Yes 2. No

38. If No. to 37 above, Why**?**

1. Was not present during distribution 2.Was not given during distribution

3. Have not started ANC 4. Does not have money 5. Don't not need one

39. Do you know the cost of an ITN in the market?

1.Yes 2. No

40. If yes to 37 above, how many mosquito bednets are there in this household?____

41. Where/on what occasion did you get it (them**).**

1. ANC 2. During a health campaign

3.Market 4. School 5. NGO 6. Red Cross

42. Do you know where your Community Treatment Unit (CTU) for MBNS is located?

1. Yes 2. No

43. If yes to question 42 above, Have you ever taken your net there for retreatment?

1. Yes 2. No

44. If yes to 43 above, was it retreated?

1. Yes **2. No**

45. If No. to 44, why? **1. CHEW not present** **2. Insecticide not available.**

3. Was charged to pay money **4. Others (specify)__________________**

46. How many of them are of short duration (need to be treated every six months)?____

47. When was (were) it (they) last treated?

1. Less than six months. **2. More than six months**

48. Have they been washed after the last retreatment?

1. Yes **2. No**

48. How many long lasting insecticide nets are there (need to be treated after five years)?____

50.When did you obtain it (them)?

1.Less than five years **2. More than five years**

51. When was (were) it(they) last retreated?

1. less than three years **2. More than three years** **3. Never retreated.**

52. How many times have they been washed after the last retreatment?__________

53. What colour (s) is (are) it (they)? ____

____

54. How do you feel about the colours? **1. Very uncomfortable 2. Uncomfortable 3. Neutral 4. Comfortable 5. Very comfortable**

55. What colour do you prefer if you are not comfortable with these ones? ___________

____

56.Why would you prefer such colour? ____

____

57. What is (are) the shape(s)

1. Rectangular ________ **2. Conical___________**

58. Which of them is easier to mount? **1. Rectangular** **2. Conical**

59. Which shape do you prefer most? ____

60. What makes you prefer the said shape? __________________________________

D. UTILIZATION OF MOSQUITO BED NETS

61. Does your household use the available bed nets? **1. Yes** **2. No**

62. If yes to question 61 above, When? **1. Always** **2. Rainy Season** **3. Dry season**

63. Why do you prefer the period above? _____________________________________

64. If No. to 61 above, what are some of the reasons for not using the available nets?

____

65. Did all children under five years in this household sleep under a bednet last night?

1. Yes **2. No**

66. If no to question 65 above, why? ____

67. Did all the pregnant women in this household sleep under a bednet last night?

1. Yes **2. No**

68. If no to question 67 above, why? ___________________________________

69. Which group of people in your household should be given priority to sleep under ITN?

1**. Children under-five years** **2. Pregnant women** **3.Children older than five** **4. Father/or Mother** **5. Grand-parents.**

70. Have you experienced any problems/difficulties using ITNS/LLINS?

1. Yes **2. No**

71. If yes to question 70 above, list them ____

72. Do you know the Community Relay Agent (CRA) for malaria activities for your community? **1. Yes** **2. No.**

73. If yes to question 72 above, how many times has he visited your home since the beginning of the rainy season?

1**. Never** **2. Once** **3. Twice** **4. Thrice** **5. Four times plus**

74. What are some of the activities carried out by the CRA during home visits.
____
____

75. How do you appreciate the work of the CRA in your community?

1. Very poor. 2. Poor **3. Good** **4. Very Good** **5. Excellent.**

Questions 76-82 are pregnant women only.

76. How old is your pregnancy? __________months

77. How far is the Nearest Health unit from your home? ___________km

78. Have you registered for ANC? **1. Yes** **2. No**

79. If yes to question 78 above in what type of health facility?

1**. Public** **2. Confessional** **3. Private**

80. Did you receive an ITN at this health unit after registration?

1. Yes **2. No**

82. If No. to question 78 above, why?

1. Pregnancy still too young **2. No money**

3. Health facility too far **4. ANC not necessary**

I have exhausted my questions for you. Do you have any worries or questions?

CAN WE NOW HAVE A LOOK AT THE BEDNETS THAT YOU HAVE?

BEDNET CHECKLIST

1. How many nets have you seen in the household?_________

2. How many are hung over the bed?_______________

3. Are they properly hung? **1. Yes** **2. No**

4. Of those hung over the beds, how many are

1. Neither torn nor have holes__________

2. Torn or have holes? _________________

5. If not hung over the bed, in what condition are they?

1. Sealed in the package 2. Hung on the windows or door. 3. Just folded and placed somewhere in the room 4. Others

____

Comments

____

____

____

____

____

____

____

DATE INTERVIEW DONE **INTERVIEWER'S NAME AND SIGNATURE**

______________________ ________________________________
